# Service users’ perceptions of relevant and helpful components of an integrated care concept (ACCESS) for psychosis

**DOI:** 10.3389/fpsyg.2023.1285575

**Published:** 2023-12-18

**Authors:** Anja Christine Rohenkohl, Pia Sowada, Martin Lambert, Jürgen Gallinat, Anne Karow, Daniel Lüdecke, Friederike Rühl, Daniel Schöttle

**Affiliations:** Department of Psychiatry and Psychotherapy, University Medical Center Hamburg-Eppendorf (UKE), Hamburg, Germany

**Keywords:** assertive community treatment, integrated care, psychosis, schizophrenia, bipolar disorder, severe mental illness, patient-reported outcome

## Abstract

**Introduction:**

Psychotic disorders have a significant impact on patients’ lives and their families, and long-term treatment with individually tailored multimodal combinations of therapies is often required. Integrated care (IC) concepts such as the “Hamburg Model (ACCESS)” with a focus on psychotic disorders, includes different (therapeutic) components with pharmaco- and psychotherapy, family involvement, home treatment and the option of using a 24/7 crisis hotline. All components are offered by a therapeutically-oriented assertive community treatment (TACT) team in a need-adapted manner. So far, however, little is known about which specific components are regarded as especially relevant and helpful by the users of IC.

**Methods:**

Patients currently participating in IC completed a questionnaire as part of the continuous quality assurance study (ACCESS II) in which they were asked to rate the different components of treatment according to their relevance and helpfulness, considering the individual’s unique experiences with IC and needs in mental health care. Furthermore, they were asked to make suggestions regarding additional helpful components of treatment.

**Results:**

Fifty patients participated in this survey (23% of the patients currently participating in the IC concept). For participants, the most helpful and important factors were having the same therapist in the long-term and the 24/7 crisis telephone. Additional components suggested by patients included more addiction-specific therapies and increased focus on vocational rehabilitation and integration.

**Conclusion:**

From the perspective of the users of IC, long-term care from a trusted therapist with whom there is a therapeutic relationship and the possibility to reach someone they already know from the TACT team 24/7 serves as the best basis for effective care, fostering trust, understanding, and open communication. In contrast, home treatment remains a relevant aspect of evidence-based care for people with severe mental illness, but perhaps surprisingly, is not viewed as the most important issue.

## 1 Introduction

Individuals with schizophrenia, bipolar disorder, and major depression with psychotic symptoms often suffer from poor quality of life (Ruggeri et al., 2000) and lack of community integration (Lambert et al., 2010). To provide comprehensive support for individuals with severe mental illness, it is imperative to address their unique needs and offer treatment in a coordinated and integrated care (IC) system (Schöttle et al., 2013).

Patients with severe and persistent mental illness (SPMI) frequently experience a chronic course of illness, underscoring the importance of timely identification, accurate diagnosis and consequent subsequent treatment (Schöttle et al., 2013; Correll et al., 2017). Collaboration among mental health professionals, primary care physicians, and family members is essential to coordinate therapies and to identify, assess and act upon early signs and symptoms of a psychotic relapse (De Hert et al., 2011). To manage symptoms effectively, treatment plans should take a holistic approach by linking psychotherapy, pharmacological treatment, and psychosocial interventions (Correll et al., 2018; Hansen et al., 2023).

Several IC models have been developed for treating people with severe mental illness (SMI), employing diverse approaches such as Assertive Community Treatments (ACT; Sytema et al., 2007; Lambert et al., 2010; Schöttle et al., 2018), Community Mental Health Teams (CMHTs; Malone et al., 2009), and Intensive Case Management (ICM; Schöttle et al., 2013; Dieterich et al., 2017).

In the early 1970s, the Assertive Community Treatment (ACT) approach played an important role in transforming interventions for people with severe mental illness (Stein and Santos, 1998). ACT comprises an evidence-based, team-centered approach with continuous, open-end treatment. To ensure comprehensive care and support for the patient, a multidisciplinary team including, for example, psychiatrists, social workers, psychologists, nurses, etc., is available (Olfson, 1990; Marshall and Lockwood, 2011). ACT also focuses on community and family integration, fostering inclusion in the community (e.g., housing, recreational activities) and involving family members in treatment as needed (Dixon et al., 1998; Stein and Santos, 1998; Philips et al., 2001), as families can foster a supportive and understanding environment for patients (Waller et al., 2019). Additionally, case management aids patients in coordinating and organizing their services and regular home visits are offered to ensure comprehensive care tailored to individual needs (Bertelsen et al., 2008). Home visits represent an important aspect: they enable continuous treatment utilization, the minimization of barriers, and the recording of living conditions by the treatment team. Severe psychotic disorders can occasionally lead to crises and relapses. Through the development of crisis intervention plans and the establishment of counseling centers, an immediate contact person is available by telephone 24 h a day (Philips et al., 2001).

Studies on the effectiveness of IC models for patients with severe mental illness have observed an overall positive impact, e.g., on symptomatology, functioning, and quality of life (Bond et al., 2001). IC models also enhance adherence, improve the perceived quality of treatment, and facilitate access to psychiatric services (Schöttle et al., 2013; Baxter et al., 2018). In previous studies (Bond et al., 2001; Lambert et al., 2010, 2015; Schöttle et al., 2018, 2019; Rohenkohl et al., 2022; Schröter et al., 2023), ACT treatment (also as a possible component of an IC concept) has been linked to various effects. Patients, who received ACT, showed improvements in quality of life, illness severity, global functioning, performance satisfaction, and treatment adherence. The efficacy of ACT has also been confirmed in reducing relapse rates (Chien et al., 2013), involuntary admissions and treatment (Schöttle et al., 2019). Due to the variety of intervention strategies offered, the question arises: which treatments are perceived as particularly helpful and effective from the patients’ perspective. For example, research has shown that CMHTs care approach results in higher patient treatment satisfaction and contributes to decreased hospitalizations compared to standard care (Malone et al., 2009).

Over the past decades, most intervention approaches have been based primarily on experiences from experts and studies not involving the users of these systems so that it remains unclear which treatment components or combination of components are regarded as especially useful and helpful by patients. Ignoring the patient’s perspective in therapy can have adverse effects on intervention effectiveness (Glynn et al., 2006). This can lead to consequences, including low motivation, higher rates of treatment discontinuation, and medication non-adherence. Previous studies have shown that patient satisfaction with the quality of care plays a critical role in treatment outcomes (Small et al., 1965; Ware and Davies, 1983). Furthermore, to achieve improved therapy adherence, cultivating a positive therapeutic relationship between the patient and the multidisciplinary team is important. Maintaining a strong therapeutic relationship over an extended period positively affects the patient’s outcomes (Holzinger et al., 2002). Thus, considering the patient’s perspective represents a crucial factor in psychiatric care. In addition to providing valuable insights into the needs, preferences, and experiences of those who need mental health care, involving patients in the assessment process can also help in continuously enhancing the quality of mental health care. By tailoring treatment plans to individual needs, mental and psychosocial health status, best possible satisfaction with treatment as well as long-term effectiveness can be achieved.

This study focuses on the patient perspective regarding which components of care users of the “Hamburg Model of Integrated Care (ACCESS)” with an adapted ACT concept perceive as being most effective and helpful. The effectiveness and efficiency of the ACCESS model was assessed in three studies: the ACCESS I study (Lambert et al., 2010) assessed the implementation of the model; the ongoing ACCESS II study (Schöttle et al., 2018; Ruppelt et al., 2020) assesses all patients entering the model; the ACCESS III study (Lambert et al., 2018) evaluated the effectiveness of the expansion of the model to adolescents (from the age of 12 years) and young adult patients in the early stage of the illness. In this current patient-based evaluation, data from the ongoing ACCESS II-study were used.

## 2 Materials and methods

In order to examine the most important elements of care from a user perspective, in the first step, a questionnaire including all care modules of IC provided by the “Hamburg Model of Integrated Care (ACCESS)” was developed. In a second step, this questionnaire was presented to all practitioners of the two ACT teams with the request to complete it and comment on it. Drawing from Delphi survey methodology, the questionnaire was adapted and, through an iterative process, the expertise of the multi-professional teams was used to improve and finalize the questionnaire to reflect all areas of care (see Methods for content of the questionnaire). In the present study, the questionnaire was then presented to ACCESS users (e.g., patients) with the request to rate the items according to helpfulness and relevance of intervention components. Moreover, participants were also asked to note if intervention components were missing from the questionnaire.

### 2.1 Study design and sample

The “Hamburg Model of Integrated Care” (ACCESS II study) is a prospective, long-term study of an IC model for people with severe psychotic disorders (non-affective and affective). The model includes Therapeutic Assertive Community Treatment (TACT) within a cross-sectoral and interdisciplinary network of inpatient and outpatient services from the adult psychiatry clinic of the University Medical Center Hamburg-Eppendorf as described in detail in previous articles (Lambert et al., 2010; Karow et al., 2012; Schöttle et al., 2014, 2018; Rohenkohl et al., 2022).

From May 2007 to November 2021, 433 patients had been treated in the continuing *“Hamburg Model of Integrated Care (ACCESS).”* As part of the ACCESS study, which represents an ongoing evaluation of the Hamburg model, trained raters evaluate the effects of treatment on symptom burden, functional level and severity of the disease at fixed intervals at the beginning of treatment and every 6 months thereafter. In addition, quality of life and satisfaction with treatment are assessed from the patients’ perspective. Because every patient in IC is approached for the ongoing evaluation within 6 months, a time interval of 6 months was selected for this survey. Patients who participated in the IC model between November 2021 and April 2022 were potentially eligible to participate in this voluntary and additional survey on patient-oriented components of care. The ACCESS trial was approved by the local ethics committee (number: PV4059) and is registered at ClinicalTrials.gov (identifier: NCT01888627). The additional survey was approved by the Local Psychological Ethics Committee at the Center for Psychosocial Medicine of the University Medical Center Hamburg – Eppendorf (number: LPEK-0379) and EmPeeRie (Empower Peers to Research) as a user-oriented science advisory service.

### 2.2 Inclusion and exclusion criteria

The eligibility criteria for participation in the ACCESS II study are as follows: Individuals (a) aged 12 years or above, (b) diagnosed with a severe psychotic disorder [Diagnostic and Statistical Manual of Mental Disorders (DSM-IV-TR): schizophrenia, schizophreniform disorder, schizoaffective disorder, delusional disorder, psychotic disorder not otherwise specified, bipolar disorder with recent severe psychotic symptoms, and major depression with severe psychotic symptoms], (c) with a symptom load indicated by a BPRS score of ≥40. (d) providing written informed consent (for participants aged 18 years or older) or written informed assent from a parent or legal guardian (for patients aged 12–17 years). Exclusion criteria included diagnoses like alcohol- or substance-induced psychosis, except when accompanied by comorbid alcohol or substance abuse or dependence, psychotic disorder resulting from a medical condition, and mental disability.

### 2.3 Assessments and measures

The questionnaire on relevant treatment impact factors recorded from the perspective of the participating patients included six scales (The questionnaire can be obtained in German from the corresponding author on request.): (1) Concept, treatment philosophy and attitude (15 items, e.g., “There are fixed reference therapists who are approachable and responsible for me.”); (2) Multi-professional teams (5 items, e.g., “Due to the availability of doctors in the team, it is easier to take or try out a medication because I can consult them at short notice.”); (3) On-call telephone – 24/7 availability of the team (2 items, e.g., “In case of a crisis, I can always reach someone by phone, even outside office hours and at weekends.”); (4) Home treatment/mobility (3 items, e.g., “The reference therapist/TACT Team will come to my home if needed/in crisis.”); (5) Crisis & inpatient stay (7 items, e.g., “My reference therapist/IV team is also there for me in case of forced/compulsory admission.”; “Through Integrated Care, placement (and coercion) can be avoided.”); and (6) Network [4 items, “The reference therapist/IC team exchanges information with all persons/institutions involved in the treatment (exchange/networking).”]. In total, the 36 items are answered on a graded Likert scale with the overarching question of how helpful and important patients perceive the content of the item to be for their psychiatric health care in the context of IC. Specifically, the question is: “How helpful and important is/would this item be for you”: (1) very helpful & important (2) somewhat helpful & important (3) neither helpful nor important (4) not helpful & important (5) not at all helpful & important (6) The statement is not applicable. At the end, participants were also asked for comments and additions (Open text field: “Comments, additions - is something still missing?”) and “What are the most helpful and important treatment offers in IC for you personally?”

In addition, socio-demographic variables [age, gender, first or multiple episode(s) of psychosis, diagnosis (affective versus non-affective psychosis)] were collected at the time of admission to the IC concept. Clinical outcome variables, such as level of functioning [Global Assessment of Functioning Scale (GAF); American Psychiatric Association, 2000], symptom burden [Brief Psychiatric Rating Scale (BPRS); Overall and Gorham, 1962], and severity of illness [Clinical Global Impressions Scale-Schizophrenia (CGI-S); Haro et al., 2003] were also recorded through the simultaneous regular evaluation.

### 2.4 Statistical analyses

A descriptive statistical analysis of the collected data on the impact factors from the perspective of participating patients was carried out. This includes mean (*M*) and standard deviation (*SD*). All analyses were carried out using SPSS, Version 27.0 (IBM Corp, 2020).

## 3 Results

### 3.1 Sample characteristics

Patients who participated in the IC model between November 2021 and April 2022 (*N* = 218) were potentially eligible to participate in this voluntary and additional survey on patient-oriented components of care. Fifty patients (23%) agreed to complete the additional and voluntary survey on the evaluation of treatment components and filled out the questionnaire completely. Sociodemographic and clinical characteristics of the final sample are displayed in Table 1. The gender ratio was balanced, also the ratio between affective and non-affective psychotic illness was similar distributed. According to clinical variables obtained at the time of the survey administration, most participants (74%) had already had several psychotic episodes, all patients had moderate to high scores for psychopathology [BPRS: *M* (*SD*) = 43.10 (15.67)] and severity of illness [CGI-S: *M* (*SD*) = 3.96 (0.90)], and a lower level of functioning [GAF: *M* (*SD*) = 59.86 (12.19)]. On average, participants had been in IC for 70.38 months (range 1 to 170 months).

**Table 1:** Clinical and sociodemographic characteristics.

Sociodemographic characteristics		*N*	Mean	*SD*
Age (years)		50	39.58	12.61
Duration in IC (months)		50	70.38	49.94
Gender	female	25		
	male	25		
Clinical characteristics				
Type of psychosis	affective	23 (46%)		
	non-affective	27 (54%)		
No. of Episodes	first	13 (26%)		
	multiple	37 (74%)		
CGI		49	5.33	0.85
GAF		49	40.39	19.96
BPRS		48	74	15.61

IC, Integrated CARE; BPRS, Brief Psychiatric Rating Scale; CGI, Clinical Global Impression Scale; GAF, Global Assessment of Functioning Scale (0–100) (Ruggeri et al., 2000; Lambert et al., 2010; De Hert et al., 2011; Schöttle et al., 2013; Correll et al., 2017, 2018; Hansen et al., 2023); informations (Exception: Duration in IC) were assessed at the time of admission to the IC concept.

### 3.2 Results of the service users’ perceptions of relevant and helpful components of IC

With regard to the most helpful and important treatment components, assessed from the perspective of the participants, the components are listed in Table 2.

**Table 2:** Relevant and helpful components of integrated care.

	M (*SD*)	Scale Content	Item*
1	1.02 (0.14)	Concept, treatment philosophy and attitude	There are **fixed reference therapists** who are **approachable & responsible** for me.
2	1.02 (0.14)	On-call telephone – 24/7 availability of the team	In case of a crisis, I can **always reach someone by phone**, even outside office hours and on weekends.
3	1.04 (0.20)	Concept, treatment philosophy and attitude	I have a **trusting relationship** with my reference therapist.
4	1.06 (0.24)	Concept, treatment philosophy and attitude	**Appointments** are made **promptly**, at **short notice** and **flexibly**.
5	1.06 (0.32)	Multi-professional teams	In integrated care, **psychotherapeutic talks** are **regularly offered**.
6	1.08 (0.27)	Crisis & inpatient stay	In the event of an inpatient admission, **UKE** is the **responsible hospital**.
7	1.10 (0.36)	Crisis & inpatient stay	**Relapse** can **be prevented** through integrated care.
8	1.12 (0.48)	Concept, treatment philosophy and attitude	The **frequency** and **duration of the appointments** can be adjusted **according to need**.
9	1.14 (0.40)	Concept, treatment philosophy and attitude	My **reference therapist** can be **easily reached**.
10	1.18 (0.44)	Concept, treatment philosophy and attitude	I feel comfortable to **talk about critical issues** (e.g., suicidal thoughts).
11	1.18 (0.77)	Crisis & inpatient stay	Integrated care can **shorten a crisis**.
12	1.18 (0.44)	On-call telephone – 24/7 availability of the team	I can also **contact the IV team at any time** when the practitioner is on holiday.
13	1.20 (0.40)	Concept, treatment philosophy and attitude	The **treatment** takes place at **eye level**.
14	1.22 (0.79)	Crisis & inpatient stay	Integrated care **can avoid/reduce inpatient admission**.
15	1.24 (0.77)	Concept, treatment philosophy and attitude	There is **no fixed end to treatment** in IV.
16	1.27 (0.45)	Multi-professional teams	The **teams are multi-professional**, i.e., doctors, psychologists, recovery counselors and social workers are available.
17	1.41 (1.10)	Multi-professional teams	The **availability of doctors** in the team makes it **easier to take or try out a medication** because I can also consult them at short notice.
18	1.51 (1.21)	Network	The reference therapist/the IV team **exchanges information with all persons/institutions** involved in the treatment (exchange/networking).
19	1.53 (1.40)	Concept, treatment philosophy and attitude	I feel **confident** enough to **openly discuss** things like a lower dose or stopping the medication on my own.
20	1.61 (1.53)	Crisis & inpatient stay	Integrated care **can avoid hospitalization** (and pressure).
21	1.69 (1.27)	Crisis & inpatient stay	For me, it is important that there is a **crisis plan**.
22	1.70 (1.27)	Network	The reference therapist/the IV team **coordinates important appointments** during treatment.
23	1.73 (1.30)	Home treatment/mobility	The reference therapist/the IV team **comes to my home** if necessary/in the crisis.
24	1.78 (4.57)	Concept, treatment philosophy and attitude	The reference therapist/the IV team **knows me over a longer period of time/the crisis**.
25	1.80 (1.17)	Multi-professional teams	I have the **option of contacting a social worker**.
26	1.82 (1.62)	Crisis & inpatient stay	My **reference therapist**/the IV team is also **there for me** in the case of placement/forced hospitalization.
27	1.86 (1.43)	Home treatment/mobility	The reference therapist/the IV team also has the **option of arranging meetings** with me **outside the UKE**.
28	1.87 (1.65)	Concept, treatment philosophy and attitude	**Addiction/consumption** and failure to keep appointments **do not lead to exclusion from IV**.
29	1.92 (1.59)	Network	**Examination** and **diagnosis of somatic** (physical) **diseases** is **coordinated** by the IV.
30	2.22 (1.93)	Concept, treatment philosophy and attitude	I feel **confident** enough to **talk openly about my consumption** (e.g., alcohol, drugs).
31	2.52 (2.18)	Network	The reference therapist/the IV team **is linked to/in exchange with the ward**.
32	2.53 (1.68)	Multi-professional teams	There is the opportunity to **exchange ideas with recovery counselors**.
33	2.62 (6.50)	Concept, treatment philosophy and attitude	The **reference therapist**/the IV team is **responsible for me** on the ward, as a day patient and as an outpatient.
34	2.98 (4.73)	Concept, treatment philosophy and attitude	There is the **possibility of couple/family therapy**.
35	3.02 (1.77)	Home treatment/mobility	The reference therapist/the IV team has the **opportunity to accompany me to network meetings**.
36	3.70 (6.41)	Concept, treatment philosophy and attitude	There is **low-threshold access to other services** in the building.

*items were rated on a Likert scale: (1) very helpful & important (2) somewhat helpful & important (3) neither helpful nor important (4) not helpful & important (5) not at all helpful & important (6) The statement is not applicable; Items were freely translated into English; the original questionnaire is available in German on request from the corresponding author.

The answers that were rated most strongly in terms of relevance in care by the user’s perspective were, “There are fixed reference therapists who are approachable & responsible for me.,” “In case of a crisis, I can always reach someone by phone; even outside office hours and at weekends.,” “I have a trusting relationship with my reference therapist.” (See Table 2). Home treatment as a core component of the *Hamburg Model of IC* was rated as a helpful and important component, but is not counted among the most relevant components (*M* = 1.73; *SD* = 1.30). Treatment elements related to network management were rated as least helpful and important by patients [e.g.” The reference therapist/the IV team is linked to/in exchange with the ward.” (*M* = 2.52; *SD* = 2.18); “The reference therapist/the IV team has the opportunity to accompany me to network meetings.” (*M* = 3.02; *SD* = 1.77)].

In response to the open question, “What is most important to you as a service user in IC?,” no new topics or treatment modules were mentioned that were not already included in the questionnaire (e.g., “therapeutic relationship,” “one-on-one talks,” “24/7 accessibility”). For the question on what is missing in the existing IC concept, more focus on the treatment of addiction as well as more support for vocational therapies were mentioned. Additional components in the IC concept that were not recorded in the questionnaire were not listed.

## 4 Discussion

Patients with non-affective and affective disorders treated in the ACCESS model of IC are offered a wide range of therapies focusing on psychopharmacological, psychotherapeutic and psychosocial treatment delivered by the TACT teams. To our knowledge, there is no study asking the users of an IC system which components of treatment they experience as most helpful and important.

The answers to the relevant treatment components from the perspective of patients as service users clearly focus on the continuous long-term relationship with their assigned therapist and the TACT team. In our teams in IC, the same therapist coordinates and conducts therapy regardless of which intensity of treatment the patient needs (e.g., inpatient, day clinic or outpatient). Within the team, the primary assigned therapist has a co-therapist who the patient knows well and who acts as a substitute for the assigned therapist in case of holidays or sickness. Developing and maintaining a trustful and stable alliance is of utmost importance, particularly when long-term treatment is necessary. Working with the same therapist over the long-term can help foster this alliance, which is particular important when working with patients with severe mental illnesses (Davis and Lysaker, 2007; Priebe et al., 2011; Goldsmith et al., 2015; Shattock et al., 2018; Browne et al., 2019; Hasson-Ohayon et al., 2019) as a strong working alliance has been shown to promote insight and improve recovery functional status and medication adherence. In our ACCESS model of IC, we had low service disengagement rates (Schöttle et al., 2013; Lambert et al., 2015, 2017; Schöttle et al., 2018, 2019). Therefore, we speculate (Lambert et al., 2017) that the clinically meaningful effects were mainly a result of the highly intensive and need-adapted IC interventions primary conducted by the same interdisciplinary TACT-Team with a focus on high quality psychopharmacological and psychotherapeutic treatment. Although we can only make assumptions as we also did not measure the therapeutic alliance itself and only asked how important it is for the service users, results of this study corroborate our hypotheses that the positive impact of our ACCESS model could at least partly be associated with the intensive and strong therapeutic alliance developed during the intensive treatment in IC.

Earlier research has demonstrated a favorable connection between therapeutic alliance and medication adherence (McCabe et al., 2012; Misdrahi et al., 2012; Shattock et al., 2018; Browne et al., 2019; Hsieh et al., 2022). Patients who demonstrated poor adherence over a period of 3 months were inclined to assign lower ratings to their perceived therapeutic alliance, unlike patients who adhered more consistently to their prescribed medication regimen (Lincoln et al., 2016). This suggests that a strong therapeutic alliance could significantly contribute to improving medication adherence. The 24/7 accessibility of a therapist they know in a crisis is also an important treatment component that is mentioned. This component can also be seen as a continuation of the therapeutic relationship. Treatment elements related to network management in IC were rated as least helpful and important because it is likely to have the last direct impact and effect for patients.

The results of this study show that obtaining the patient perspective can provide unique information on quality of care. While there is good evidence for ACT treatment in severe mental illness in terms of clinical outcomes (Bond et al., 2001; Lambert et al., 2010, 2015; Chien et al., 2013; Schöttle et al., 2018, 2019; Rohenkohl et al., 2022), it is not mentioned by participating patients as one of the most relevant components of the “Hamburg Model” care concept.

Long-term treatment in psychiatry often involves establishing a therapeutic relationship between the patient and the mental health professional. This relationship serves as a foundation for effective care, fostering trust, understanding, and open communication. Through ongoing sessions and interventions, the therapeutic relationship allows for the exploration of deeply rooted issues, the development of coping strategies, and the gradual progress toward mental well-being. The continuity of this relationship over an extended period enables the patient to work through challenges, gain insights into their condition, and achieve lasting positive changes in their emotional and psychological state (Adair et al., 2005; Catty et al., 2013; Puntis et al., 2015; de Cruppé et al., 2023).

In summary, this preliminary study gives an indication that the effectiveness and efficiency of psychiatric and psychotherapeutic care for people with psychosis is not solely a question of guidelines and economics. A triad consisting of patient perspective, guidelines (evidence level) and economic perspective (cost-effectiveness) can thus best answer the question of the best possible care for people with severe mental illness.

## 5 Limitations and outline

During the COVID-19 pandemic, much of the treatment was also shifted to the telephone or meetings outside the clinic. This is one possible reason why only about 25% of eligible participants participated in this survey. Some of the interviews of the regular quality assurance survey were also conducted by telephone but this additional survey was not due to the length of the questionnaire, which means that some patients were likely not asked to participate at all.

According to this user survey, the existing IC concept should be slightly adapted to include additional addiction-specific interventions, as well as more counseling in the direction of supported employment and education. To address this topic, there is an ongoing study investigating whether targeted job coaching during the early stages of psychosis (first 5 years of illness) has an impact on participation in the primary job market (Jäckel et al., 2023). Results might aid to implement the field of supported employment as an additional component in the care of individuals with severe psychotic disorders, e.g., within the IC concept. To address comorbid substance use disorders more effectively, it is essential to further enhance the connectivity with outpatient resources, such as addiction counseling centers. This expansion can encourage the regular utilization of services offered from these centers and lower the barrier for individuals to seek assistance from them.

To further focus on this topic and continue involving patients, it should be investigated on a larger sample whether there are, e.g., differences in needs per diagnostic group (affective versus non-affective psychosis) or age group. In additional analyses with all participating patients, it should be considered which treatment components have an influence on clinical outcomes, quality of life and satisfaction with care.

### 5.1 Clinical implications

The findings speak to the need for the implementation and promotion of long-term approaches in the care of people with severe mental illness (psychosis). Additionally constant accessibility serves as relevant component of care to avoid crises and optimize care.

Thus, a patient-centered approach to mental health care provision should be fostered, aiming to enhance quality of life and empowering patients to take a more active role in their course of treatment. Furthermore, a patient-centered assessment allows for greater consideration of patient’s individual needs within a care framework, thereby addressing gaps in provision. Additionally, patient concerns consistently serve as compelling incentives to tailor and enhance mental health services.

## Data availability statement

The raw data supporting the conclusions of this article will be made available by the authors, without undue reservation.

## Ethics statement

The studies involving humans were approved by the local ethic committee approved the ACCESS study (registration number: PV4059). The study was registered at ClinicalTrials.gov (identifier: NCT01888627). This survey was approved by the Local Psychological Ethics Committee at the Center for Psychosocial Medicine of the University Medical Center Hamburg – Eppendorf (number: LPEK-0379) and EmPeeRie (Empower Peers to Research) as a user-oriented science advisory service. The studies were conducted in accordance with the local legislation and institutional requirements. The participants provided their written informed consent to participate in this study.

## Author contributions

AR: Conceptualization, Formal analysis, Methodology, Project administration, Writing – original draft, Writing – review & editing. PS: Writing – original draft, Writing – review & editing. ML: Conceptualization, Investigation, Supervision, Writing – review & editing. JG: Writing – review & editing. AK: Writing – review & editing. DL: Writing – review & editing. FR: Writing – review & editing. DS: Conceptualization, Methodology, Supervision, Writing – original draft, Writing – review & editing.
